# Malnutrition and Associated Factors in Acute and Subacute Stroke Patients with Dysphagia

**DOI:** 10.3390/nu15173739

**Published:** 2023-08-26

**Authors:** Jiyong Yoon, Soyeong Baek, Yunjeong Jang, Chang Han Lee, Eun Shin Lee, Hayoung Byun, Min-Kyun Oh

**Affiliations:** 1 Department of Rehabilitation Medicine, Gyeongsang National University College of Medicine and Gyeongsang National University Changwon Hospital, Changwon 51472, Republic of Korea; nini002@naver.com (J.Y.); soyb1024@gnuh.co.kr (S.B.); jyjer@naver.com (Y.J.); ychkhk1407@gmail.com (C.H.L.); 2 Department of Rehabilitation Medicine, Gyeongsang National University College of Medicine and Gyeongsang National University Hospital, Institute of Medical Science, Gyeongsang National University, Jinju 52727, Republic of Korea; rmeslee@gnu.ac.kr; 3 Department of Rehabilitation Medicine, Gyeongsang National University College of Medicine and Gyeongsang National University Hospital, Jinju 52727, Republic of Korea

**Keywords:** malnutrition, body weight, albumin, stroke, dysphagia, VFSS, age, mRS, MMSE, pneumonia

## Abstract

Most patients with stroke suffer from complications and these include dysphagia. Dysphagia can cause malnutrition, and malnutrition affects prognosis and recovery. However, there is a lack of accurate studies on the nutritional status of stroke patients with dysphagia and its associated factors in different phases of stroke. This study retrospectively investigated 620 stroke patients who underwent a videofluoroscopic swallowing study (VFSS) due to dysphagia, from March 2018 to February 2021. The study aimed to evaluate the nutritional state and associated factors of malnutrition in acute and subacute stroke patients with dysphagia. Serum albumin and percentage of current weight to ideal weight were used to determine nutritional status. Malnutrition was observed in 58.9 and 78.9% of acute and subacute stroke patients. Exact logistic regression analysis revealed that old age and high penetration–aspiration scale score were significantly associated factors for malnutrition in patients with acute stroke. Old age, stroke history, bilateral hemiplegia, high modified Rankin score, low Korean Mini-Mental State Examination, pneumonia, and high functional dysphagia score were significantly associated factors for malnutrition in patients with subacute stroke. Patients with these associated factors in each phase of stroke require active nutritional assessment and care to decrease the risk of malnutrition.

## 1. Introduction

Stroke is one of the leading causes of death and disability, and its burden is increasing with time [1]. Complications are one of the reasons for this burden, and stroke complications exist in 40 to 95% of patients [2]. Major complications after stroke include dysphagia, seizure, pneumonia, urinary tract infection, gastrointestinal disorders, deep vein thrombosis, pain, depression and pulmonary embolism [2,3,4]. Dysphagia is one of the major problems in patients with acute stroke, and it affects more than 50% of stroke survivors [5]. Most patients with dysphagia in the acute stroke phase recover swallowing function within a week, but some patients have prolonged dysphagia [6,7]. Dysphagia affects the ability to swallow safely and efficiently, and causes difficulties in consuming an adequate and balanced diet [8]. Therefore, dysphagia after stroke can cause malnutrition, aspiration pneumonia, and dehydration and affects the quality of life [5,9]. Malnutrition after stroke occurs in 6 to 62% of patients [10]. Malnutrition increases the risk of infection and complications, and has an impact on the length of hospital stay, functional recovery, and prognosis after stroke [10]. Therefore, nutritional status evaluation in patients with stroke is important to provide appropriate nutritional interventions to overcome the consequences of stroke and to reduce the risk of malnutrition.

Old age, gender, dysphagia, poor nursing care, delayed rehabilitation, chronic diseases such as diabetes mellitus, hypertension and depression, previous severe alcoholism, stroke history, cognitive impairments, and functional disabilities are associated factors for malnutrition after stroke [10]. However, the impact of each factor may differ depending on the duration of the stroke. In the early phases of a stroke, the acute disease state itself and the polypharmacy used to control it affect malnutrition, and the complications thereafter affect malnutrition [11]. Additionally, some factors that affect nutritional status, such as dysphagia, depression, cognitive impairments and functional disabilities may change during stroke phases. Several studies attempted to determine the associated factors of malnutrition after stroke in acute and subacute phases. Finestone et al. revealed tube feeding, diabetes and prior stroke as the significantly associated factors of malnutrition on admission, and advanced age at follow up [12]. Axelsson et al. found that female sex, older age, and arterial fibrillation history were associated with nutrition on admission. In contrast, male sex, infections and cardiovascular drugs were associated with nutrition at discharge [13]. The study by Foley et al. revealed that the overall odds of malnutrition in patients with dysphagia were 2.425, which were higher during the rehabilitation phase (odds ratio (OR): 2.445) than during the first 7 days of stroke onset (OR: 2.401) [14].

Identifying the nutritional status of stroke patients is important to reduce the risk of malnutrition. However, subjective assessment of nutritional status is not possible in some stroke patients due to medical condition or cognition. Also, it is important to understand the identifying factors associated with malnutrition to reveal the complex interaction of the various elements that contribute to malnutrition. In addition, understanding the factors associated with malnutrition would help to develop the evidence-based interventions to treat malnutrition. However, we may need to focus on different factors at different phases of stroke, because patients may differ in medical condition or feeding method. Identifying the most influential factors at each phase may help to determine which patients to focus on to improve nutritional status. To the best of our knowledge, few studies have analyzed the detailed nutritional status of stroke patients with dysphagia and associated factors of malnutrition at different stroke phases. Therefore, this study aimed to analyze the various nutritional statuses of stroke patients with dysphagia in acute and subacute phases, using anthropometry and laboratory examinations, which are objective measurements. We used these measurements because these factors are readily available through electronic medical records. Therefore, quick and broad assessment of nutritional status would be possible in patients without additional subjective assessments. The study also aimed to identify the associated factors at the acute and subacute phases of stroke, and to find the most significantly associated factor for malnutrition in each phase. Patients with these significantly associated factors should undergo active nutritional assessment and care to improve the nutritional status.

## 2. Materials and Methods

We retrospectively reviewed the medical records for stroke patients who visited Gyeongsang National University Jinju and Changwon Hospital from March 2018 to February 2021 for a VFSS due to dysphagia. Demographic data (age, gender, height, and weight), stroke-related profiles (onset of stroke, stroke type, brain-lesion location, hemiplegic side, and stroke history), functional and cognition assessment scales (modified Barthel Index (MBI), Brunnstrom stage, Functional Ambulation Category (FAC), modified Rankin score (mRS), and Korean Mini-Mental State Examination (K-MMSE)), medical history (hypertension, diabetes mellitus, dyslipidemia, alcoholism), post-stroke complications (shoulder pain, post-stroke depression, stroke progression, gastrointestinal disorders, electrolyte abnormality, seizure, urinary catheterization, urinary tract infection and pneumonia), VFSS results (functional dysphagia scale (FDS) and penetration–aspiration scale (PAS)), dietary state and nutritional status (serum albumin and percentage of current body weight to ideal body weight) were collected for analysis.

Based on adult International Classification of Diseases, Ninth Revision, Clinical Modification (ICD-9 CM), we classified nutritional status into six nutritional state groups using serum albumin level and body weight [15]. We used the Hamwi method to calculate the ideal body weight. We defined normal nutritional when serum albumin level was >35 g/L and current body weight was >90% of ideal body weight. We defined mild malnutrition when serum albumin level was >35 g/L and the current body weight was between 76 and 90% of the ideal body weight. We also defined mild malnutrition when serum albumin level was between 31 and 35 g/L and the current body weight was ≥76% of the ideal body weight. We defined moderate malnutrition when serum albumin was between 25 and 30 g/L, and the current body weight was between 60 and 90% of the ideal body weight. We also defined moderate malnutrition when albumin level was between 31 and 35 g/L and the current body weight was ≤75% of the ideal body weight. We also defined moderate malnutrition when serum albumin was <25 g/L and the current body weight was between 76 and 90% of the ideal body weight. We defined energy malnutrition (marasmus) when serum albumin was >35 g/L and current body weight was ≤75% of the ideal body weight. We defined protein malnutrition (kwashiorkor) when serum albumin was ≤30 g/L and current body weight was >90% of the ideal body weight. We defined severe protein–energy malnutrition when serum albumin was <25 g/L and current body weight was ≤75% of the ideal body weight. We also defined severe protein–energy malnutrition when serum albumin was between 25 and 30 g/L and current body weight was <60% of the ideal body weight.

We compared clinical characteristics between patients with stroke of acute and subacute phases. Before the Student *t*-test, we conducted the Shapiro–Wilk test to confirm the normality of the sample. We assessed the continuous variables with normal distribution using the Student’s *t*-test, and Mann–Whitney test for those without normal distribution. We used chi-square test to analyze the categorical variables. We conducted the logistic regression analysis to address all variables as associated factors of malnutrition for total and subacute stroke patients. We conducted an exact logistic regression analysis for acute stroke patients due to the small number of subjects. We used the Statistical Package for the Social Sciences version 21.0 (IBM SPSS, Armonk, NY, USA) and Stata version 18.0 (StataCorp LLC, College Station, TX, USA) for statistical analysis. We considered a statistically significant difference at *p*-values of <0.05.

## 3. Results

Among the 620 patients, 107 (17.3%) had had an acute stroke, and 513 (82.7%) had had a subacute stroke. Age, gender distribution, stroke type, brain-lesion location, hemiplegic side and proportion of patients with stroke history were not significantly different between the two groups. The mean stroke duration was 4.97 ± 1.80 days in acute stroke patients, and 28.6 ± 25.3 days in subacute stroke patients (Table 1).

### 3.1. Nutritional Status of Acute and Subacute Stroke Patients with Dysphagia

**Figure 1:**
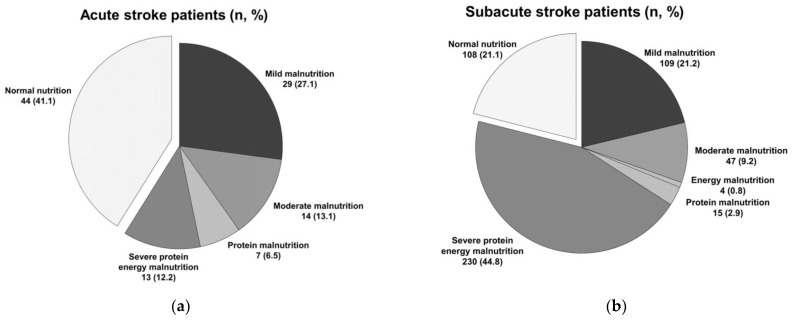
(**a**) Nutritional status of acute stroke patients with dysphagia; (**b**) nutritional status of subacute stroke patients with dysphagia.

Of the patients, 41.1 and 21.1% with acute and subacute stroke, respectively, showed normal nutrition (Figure 1). Additionally, 58.9 and 78.9% of patients with acute and subacute stroke, respectively, demonstrated malnutrition. Subacute stroke patients were more likely to have malnutrition status than acute stroke patients (OR: 2.62; 95% confidence interval (CI): 1.69–4.07; *p* = 0.000). We found mild malnutrition in 27.1 and 21.2% of patients with acute and subacute stroke, respectively. We found a moderate malnutrition state in 13.1 and 9.2% of patients with acute and subacute stroke, respectively. We found an energy malnutrition state in 0 and 0.8% of patients with acute and subacute stroke, respectively. We found a protein malnutrition state in 6.5 and 2.9% of patients with acute and subacute stroke, respectively. We found severe protein–energy malnutrition in 12.2 and 44.8% of patients with acute and subacute stroke, respectively (Figure 1). Therefore, this study found that malnutrition was more common in subacute stroke patients with dysphagia than acute stroke patients. Also, severe protein–energy malnutrition was the most common nutritional status in subacute stroke patients with dysphagia.

### 3.2. Associated Factors for Malnutrition in Stroke Patients with Dysphagia

Among the variables, we found a significant relationship between malnutrition and age (OR: 1.11; 95% CI: 1.08–1.14; *p* = 0.000), stroke duration (OR: 1.03; 95% CI: 1.01–1.06; *p* = 0.011), stroke history (OR: 4.57; 95% CI: 1.90–11.0; *p* = 0.001), bilateral hemiplegia (OR: 2.62; 95% CI: 1.09–6.27; *p* = 0.031), Brunnstrom stage of the proximal upper extremity (OR: 0.79; 95% CI: 0.65–0.96; *p* = 0.020), mRS (OR: 1.42; 95% CI: 1.17–1.73; *p* = 0.001), K-MMSE score (OR: 0.95; 95% CI: 0.92–0.98; *p* = 0.002), presence of pneumonia (OR: 2.26; 95% CI: 1.20–4.27; *p* = 0.012), the results of FDS (OR: 1.02; 95% CI: 1.00–1.04; *p* = 0.008), and PAS (OR: 1.13; 95% CI: 1.02–1.24; *p* = 0.016) (Table 2). Also, we found an association between malnutrition and the presence of stroke progression and electrolyte abnormality, but this was not statistically significant (Table 2). Stroke history was the most significantly associated factor for malnutrition in patients with acute and subacute stroke. Therefore, active nutritional assessment and care would be recommended for stroke patients with a history of stroke.

### 3.3. Differences in Associated Factors between Acute and Subacute Stroke Patients with Dysphagia

We conducted further analysis in each group to determine whether significant variables were the same in acute and subacute stroke patients. Exact logistic regression analysis revealed that age (OR: 3.05; 95% CI: 1.68–4.84; *p* = 0.000), and PAS result (OR: 1.27; 95% CI: 0.20–2.44; *p* = 0.018) were significantly associated factors of malnutrition in acute stroke patients (Table 3). In subacute stroke patients, age (OR: 1.11; 95% CI: 1.08–1.15; *p* = 0.000), stroke history (OR: 6.25; 95% CI: 1. 80–21.8; *p* = 0.004), bilateral hemiplegia (OR: 3.14; 95% CI: 1.08–9.11; *p* = 0.035), mRS (OR: 1.54; 95% CI: 1.21–1.95; *p* = 0.000), K-MMSE score (OR: 0.94; 95% CI: 0.91–0.98; *p* = 0.001), pneumonia (OR: 2.17; 95% CI: 1. 07–4.38; *p* = 0.032), and FDS results (OR: 1.03; 95% CI: 1.01–1.05; *p* = 0.008) were significantly associated factors of malnutrition (Table 3). We found an association between malnutrition and stroke duration, Brunnstrom stage of the proximal upper extremity, stroke progression, electrolyte abnormality, and the result of the PAS, but it was not statistically significant (Table 3). Age was the most significantly associated factor for malnutrition in acute stroke patients. In addition, stroke history was the most significantly associated factor for malnutrition in subacute stroke patients. Therefore, we need to focus on the nutritional status of elderly patients in the acute stroke phase, and patients with a stroke history in the subacute stroke phase.

## 4. Discussion

This study aimed to investigate the nutritional status of stroke patients with dysphagia in the acute and subacute phases. It also aimed to determine the associated factors of malnutrition in each phase. We found that 58.9 and 78.9% of patients with acute and subacute stroke were malnourished. Subacute stroke patients were more likely to be malnourished than acute stroke patients, with an OR of 2.62. We also found that severe protein–energy malnutrition was most common in subacute stroke patients. Therefore, subacute stroke patients require more active nutritional assessments and care compared to acute stroke patients. Age and PAS results were significantly associated factors of malnutrition in acute stroke patients. Age, stroke history, bilateral hemiplegia, mRS, K-MMSE score, pneumonia, and FDS results were significantly associated factors of malnutrition in subacute stroke patients. Among the various factors, stroke history was the most significantly associated factor of malnutrition in stroke patients, with an OR of 4.57. Further analysis revealed that age and stroke history were the most significantly associated factors for malnutrition in acute and subacute stroke patients, with ORs of 3.05 and 6.25, respectively. Therefore, elderly stroke patients and stroke patients with a stroke history would require more active nutritional assessments and care during acute and subacute phase of stroke, respectively.

In this study, the prevalence of malnutrition increased from 58.9 to 78.9% in acute and subacute stroke patients with dysphagia, respectively. The results of our study support previous studies revealing that malnutrition tends to worsen over time after stroke. From a study with 104 stroke patients, Davalos et al. found that malnutrition was observed in 16.3% of their subjects on admission. In their study, malnutrition increased to 26.4 and 35% in the first and second week of admission after stroke, respectively [16]. From the review article with pooled analysis by Foley et al., the odds of malnutrition significantly increased during the rehabilitation phase to 2.445, but not during the first 7 days of hospital admission in stroke patients [14]. A retrospective observational study with 205 acute stroke patients by Sato et al. revealed that the prevalence of malnutrition was 42% at admission and 76% at discharge [17]. A prospective observational study with 131 acute ischemic stroke patients by Yoo et al. revealed that malnutrition increased from 12 to 20% during the first week of hospitalization [18]. Also, a similar study by Mosselman et al. showed an increase from 5 to 26% over the 10 days after hospitalization [19]. Brain injury resulting from stroke has metabolic consequences. Stroke itself may act as a catabolic disease, which alters body composition, with shrinkage of body fat and cell-mass compartments. The catabolic phase of stroke contributes to a negative energy and nutritional balance which may cause a nutritional deterioration in stroke patients [16]. Also, the body’s response to injury and recovery in stroke patients would require additional energy in response to modified carbohydrate metabolism [16]. Appropriate nutrition assessment and support would be necessary for stroke patients due to the changing metabolism and increased possibilities of some post-stroke complications. In addition, nutritional assessment shortly after stroke onset might reflect the pre-stroke nutritional state, which may be before the metabolism changes [14]. Therefore, malnutrition may increase over time after stroke onset.

However, there are other studies that revealed that duration of stroke had no significant relationship with malnutrition. The study by Finestone et al. indicated that the prevalence of malnutrition was 47% on admission, which declined to 19% at the 4-month follow up [12]. The difference may be related to the variables used in each study to define malnutrition. In particular, some anthropometric measures, such as the mid-arm circumference used in Finestone et al.’s study, may not be expected to reflect rapid changes in nutritional status. These measurements rely on physical changes that are reactive to prolonged malnutrition [20]. In addition, mid-arm circumference has low sensitivity and specificity [21]. Also, serum albumin measured within 24 h of stroke onset may not be affected by the acute stress response after stroke due to the long half-life of albumin. Alternatively, changes in serum albumin levels could be attributed to an underlying pathological process rather than nutritional status. This is because the hepatic production of many proteins is downregulated during acute illness periods [14,22]. Other laboratory parameters such as total lymphocyte count and transferrin can be affected by the presence of inflammation [10]. Finestone et al. defined malnutrition when two of the six criteria were satisfied [12]. However, such a definition may allow malnutrition only to be defined with laboratory parameters or anthropometric variables. Although there is no gold standard for nutritional status assessment, malnutrition definition requires a careful combination of different assessments. Our study would be more persuasive to find the relationship between stroke and malnutrition, since we used biochemical measurement together with anthropometric variables to determine the nutritional status.

The results of malnutrition studies may vary depending on the patient’s hospitalization status. The study by Davalos et al., Yoo et al., and Mosselman et al. investigated hospitalized patients [16,18,19]. These studies showed increasing malnutrition after stroke. Conversely, the study by Finestone et al., which showed decreasing malnutrition after stroke, investigated hospitalized and discharged patients [12]. Although our study found increasing malnutrition prevalence from acute to subacute, the study included both hospitalized and outpatients. Since the hospitalization status of the patient may influence the nutritional status, a future study, which reflects hospitalization status, will be required.

The results of our study support previous studies that reported malnutrition is common in stroke patients with a history of stroke [12,23]. Finestone et al. revealed that patients with previous strokes were 71% more likely to be malnourished on admission to rehabilitation when compared to those without previous strokes [12]. A meta-analysis by Chen et al. with 8838 participants revealed that the previous stroke increased the risk of malnutrition with an OR of 3.04 [23]. Repeated stroke episodes are correlated with more neurological deficits, regarding self-feeding impairments [23]. Therefore, stroke history would increase the incidence of malnutrition with more neurologic deficits, and this is consistent with the results of our study.

There are studies that revealed that age was significantly related to malnutrition in stroke patients. From a prospective study with 49 stroke patients, Finestone et al. revealed that advanced age (>70 years) at 1 month after stroke was significantly associated with malnutrition [12]. With 100 consecutive patients with acute stroke, Axelsson et al. found that an age of 75 years old or older was significantly associated with malnutrition in stroke patients on admission and at discharge [13]. Aging is associated with several changes that can affect nutritional status, such as loss of taste and smell, poor appetite, poor oral health and dental problems [24]. Additionally, older patients tend to be taking more medications, which causes additional impairments such as anorexia and lethargy. This may affect the ability to self-feed [12]. Patients with stroke in old age have shown a higher risk of functional dependence and poorer functional recovery. This would affect their eating ability, in addition to the natural changes of aging [25,26]. Our study also revealed that age was a significantly associated factor for malnutrition in both acute and subacute stroke phases. Furthermore, age was the most significantly associated factor for malnutrition in acute stroke patients. This may because age has a greater effect than other factors in the early phase of the stroke. Therefore, nutritional assessment and management is important in elderly patients with stroke, especially during the acute phase of stroke.

Functional disabilities such as muscle weakness and apraxia affect malnutrition by affecting feeding ability and resulting in inadequate dietary intake. Several previous studies showed this. Davalos et al. found that the Barthel Index was lower in patients with malnutrition [16]. Sabbough and Torbey also emphasized the importance of arm weakness when assessing the risk of malnutrition development [10]. In this study, bilateral hemiplegia compared to left or right hemiplegia, Brunnstrom stage of the proximal upper extremity, and mRS were significantly related to malnutrition in stroke patients. Patients with right or left hemiplegia may functionally utilize the other side for self-feeding. However, patients with bilateral hemiplegia may not be able to use either side. Some factors such as the Brunnstrom stage of lower and distal upper extremities, and FAC were not associated with malnutrition. This may because the functions related to these factors were less important for self-feeding ability. Therefore, nutritional status should be evaluated and managed in stroke patients with upper extremity weakness and disabilities that affect eating behavior.

Moreover, cognitive changes affect eating behaviors by altering concentration and forgetting the memories for eating, swallowing or chewing [27]. From a study with 344 acute stroke patients by Lee et al., malnutrition defined by serum albumin and body weight was significantly associated with lower K-MMSE and post-stroke cognitive impairment [28]. Finestone and Greene-Finestone mentioned cognitive changes as secondary factors contributing to nutritional impairments after stroke [27]. Also, a cross-sectional study with 940 patients with dementia revealed that an MMSE score with an OR of 0.95 was a significant risk factor for malnutrition [29]. This study also showed that a K-MMSE score with an OR of 0.95 was a significantly associated factor of malnutrition in acute and subacute stroke patients. Therefore, nutritional status monitoring is necessary for stroke patients with low MMSE scores or impaired cognition. However, this study used only K-MMSE for cognition evaluation. Therefore, a future study with more detailed neuropsychological evaluations would be helpful to strengthen the results of this study.

Respiratory and urinary tract infections are some of the common complications after stroke. Confinement to bed, reduced ability to cough, use of urinary catheterization and immunosuppression in post-stroke recovery may increase the risk of infections after stroke [12,16]. Any disorders including stroke and infection cause or aggravate malnutrition by altering metabolism, appetite, absorption, or the assimilation of nutrients [30]. Additionally, infection may lead to decreased nutritional intake, and increased energy and protein requirements [30]. Pneumonia may occur in approximately 10% of hospitalized stroke patients. Its incidence increases up to 40% with risk factors such as advanced age, severe stroke and the presence of dysphagia [5]. Urinary tract infection may occur in 2–27% of stroke patients following stroke [31]. From Axelsson et al.’s study, infection was the strongest single predictor of poor nutritional status [13]. A meta-analysis by Chen et al. revealed the impact of pneumonia and infection on malnutrition with ORs of 2.18 and 2.75, respectively, [23]. This study also revealed pneumonia as a significantly associated factor of malnutrition in stroke patients with an OR of 2.26. Therefore, stroke patients with respiratory infections should be monitored and supported for proper nutritional status, especially during subacute phase.

Swallowing impairment reduces oral intake, which leads to malnutrition and dehydration. Decreased deglutition safety causes choking and tracheobronchial aspiration, and thereby results in pneumonia [32]. A strong relationship between the severity of dysphagia and malnutrition has been shown by several studies. Finestone et al. revealed that dysphagia was significantly associated with malnutrition on admission to rehabilitation [12]. Clave et al. found that the clinical severity of dysphagia correlated with marasmic malnutrition [33]. Chai et al. and Chen et al. revealed the OR of dysphagia for malnutrition as 2.6 [23,34]. Davalos et al. found that the OR of swallowing disability at admission for malnutrition was 5.9 [16]. This study revealed consistent results with other studies and found that results of the PAS and FDS were significantly related to malnutrition in acute and subacute stroke patients with dysphagia, respectively. VFSS is a gold standard for the study of dysphagia in oral and pharyngeal phases, by dynamically exploring the entire swallowing process [32]. The PAS was developed to classify the severity of airway invasion during swallowing [35]. The FDS quantifies functional dysphagia by evaluating oral movement, pharynx defense mechanisms, residues and delay times [36]. Both scales are known to be reliable and correlate with each other, but each reflects different variables. Dysphagia in the acute phase would be different from the subacute phase, because the neurological condition that affects swallowing can deteriorate. Also, the acute nature of the disease may change [7]. In the study by Smithard et al., the number of stroke patients with aspiration decreased over time, from 31% on day 1 to 3% on day 28 after stroke [7]. This may support the results of our study showing that the PAS, which is the scale for aspiration, is important during the acute stroke phase. However, the PAS does not specify the amount and timing of penetration and aspiration. It also does not provide information on other measurements such as oral and pharyngeal transient times, which would be important after aspiration improved. Therefore, the results of this study indicate a need to focus on the results of the PAS in acute stroke patients with dysphagia, and the FDS in subacute stroke patients with dysphagia. Stroke patients with a high PAS score during the acute phase, and a high FDS during subacute phase would require more active nutritional assessment and care to avoid malnutrition.

## 5. Conclusions and Future Work

This study has several limitations. First, there may have been selection bias in the included patients owing to its retrospective nature. Also, the study is limited by data contained within the electronic medical records, and this could not be corroborated by assessing the actual patients. Additional important factors such as detailed dietary intake would enhance the methodology of a related future study. Moreover, the study population was limited to a specific region, because data were collected from two hospitals in the same province of an Asian country. Also, the number of subacute stroke patients outnumbered the acute stroke patients. To complement the small number of acute stroke patients, we used exact logistic regression analysis. A future prospective cohort study with more recruiting centers and more acute stroke patients using comprehensive assessments would be necessary to improve generalizability and strengthen the results of this study.

However, this study has the following strengths. The validity and accuracy of the indicators are important for nutritional assessments. This study classified nutritional status using both biochemical and anthropometric measurements used in the ICD-9 CM criteria. Additionally, the inclusion of a sufficient number of subjects in a study is important. The results of this study were enhanced in their quality, reliability and generalizability because this study included more than 600 stroke patients with dysphagia. This study included 12 variables after analyzing 27 possible associated factors. An increased number of variables may help to interpret more complex relationships and interactions between variables as actual patients. However, the number of variables included in the analysis may affect the reliability [37]. Therefore, a future study with the proper number of associated factors would be helpful.

In conclusion, this study revealed that subacute stroke patients with dysphagia were more like to be malnourished than acute stroke patients with dysphagia. Also, acute stroke patients who were elderly, or with aspiration require active nutritional assessment and care to avoid malnutrition. Additionally, it was indicated in subacute stroke patients with dysphagia, who were elderly, exhibited bilateral hemiparesis, had severe disability, impaired cognitive function, stroke history, pneumonia, and severe dysphagia, that attention should be paid to their nutritional status to identify and treat malnutrition. Among the associated factors, age in acute stroke phase and stroke history in subacute stroke phase were the most significantly associated factors. Therefore, patients with these factors at each phase would require more active nutritional assessment and care.

## Figures and Tables

**Table 1:** Comparison of demographic characteristics according to stroke onset.

Variable	Acute Stroke Patients (*n* = 107)	Subacute Stroke Patients (513)	*p*-Value
Age (years)	69.3 ± 13.4	71.3 ± 12.9	0.149
Gender			
Male (*n*, %)	57 (53.3)	293 (57.1)	0.466
Female (*n*, %)	50 (46.7)	220 (42.9)	
Stroke duration (days)	4.97 ± 1.80	28.6 ± 25.3	0.000 *
Stroke type			0.611
Ischemic (*n*, %)	72 (67.3)	332 (64.7)	
Hemorrhagic (*n*, %)	35 (32.7)	181 (35.3)	
Brain-lesion location			0.215
Cortical (*n*, %)	22 (20.6)	148 (28.8)	
Infratentorial (*n*, %)	10 (9.3)	45 (8.8)	
Subcortical (*n*, %)	75 (70.1)	320 (62.4)	
Hemiplegic side			0.453
Right (*n*, %)	42 (39.3)	235 (45.8)	
Left (*n*, %)	45 (42.0)	196 (38.2)	
Bilateral (*n*, %)	20 (18.7)	82 (15.9)	
Stroke history (*n*, %)	30 (28.0)	104 (20.3)	0.076

* denotes *p*-values < 0.05, obtained by a Student’s *t*-test.

**Table 2:** Associated factors for malnutrition in stroke patients with dysphagia.

	Coefficient (β)	*p*-Value
Age	1.11 (1.08–1.14)	0.000 *
Stroke duration	1.03 (1.01–1.06)	0.011 *
Stroke history	4.57 (1.90–11.0)	0.001 *
Bilateral hemiplegia	2.62 (1.09–6.27)	0.031 *
Brunnstrom stage (proximal upper extremity)	0.79 (0.65–0.96)	0.020 *
Modified Rankin score	1.42 (1.17–1.73)	0.001 *
Korean Mini-Mental State examination	0.95 (0.92–0.98)	0.002 *
Stroke progression	2.72 (0.84–8.81)	0.095
Electrolyte abnormality	2.37 (0.83–6.77)	0.106
Pneumonia	2.26 (1.20–4.27)	0.012 *
Functional dysphagia scale	1.02 (1.01–1.04)	0.008 *
Penetration–aspiration scale	1.13 (1.02–1.24)	0.016 *

* denotes *p*-values < 0.05, obtained by a logistic regression analysis.

**Table 3:** Differences in associated factors between acute and subacute stroke patients with dysphagia.

	Acute Stroke Patients		Subacute Stroke Patients	
	Coefficient (β)	*p*-Value	Coefficient (β)	*p*-Value
Age	3.05 (1.68–4.84)	0.000 ^†^	1.11 (1.08–1.15)	0.000 *
Stroke duration			1.02 (1.00–1.05)	0.115
Stroke history			6.25 (1.80–21.8)	0.004 *
Bilateral hemiplegia			3.14 (1.08–9.11)	0.035 *
Brunnstrom stage (proximal upper extremity)			0.82 (0.65–1.02)	0.073
Modified Rankin score			1.54 (1.21–1.95)	0.000 *
Korean Mini-Mental State Examination			0.94 (0.91–0.98)	0.001 *
Stroke progression				0.997
Electrolyte abnormality			2.70 (0.80–9.14)	0.112
Pneumonia			2.17 (1.07–4.38)	0.032 *
Functional dysphagia scale			1.03 (1.01–1.05)	0.008 *
Penetration–aspiration scale	1.27 (0.20–2.44)	0.018 ^†^	1.11 (0.99–1.25)	0.069

^†^ denotes *p*-values < 0.05, obtained by an exact logistic regression analysis. * denotes *p*-values < 0.05, obtained by a logistic regression analysis.

## Data Availability

The data presented in this study are available on request from the corresponding authors.
